# Perceived impact of contextual determinants on depression, anxiety and stress: a survey with university students

**DOI:** 10.1186/s13033-019-0275-x

**Published:** 2019-03-26

**Authors:** Nasih Othman, Farah Ahmad, Christo El Morr, Paul Ritvo

**Affiliations:** 1 School of Health Policy and Management, York University, Toronto, Canada; 2 School of Kinesiology and Health Science, York University, Toronto, Canada

**Keywords:** Depression, Anxiety, Stress, Determinants, Students, Canada

## Abstract

**Background:**

Young adults starting college or university education encounter multiple stressors related to transitional life-stage and novel environments. Current studies reveal high rates of symptoms related to common mental health problems like depression, anxiety and stress. However, limited knowledge exists on the determinants on these problems among Canadian students. The primary aim of the study was to investigate the impact of contextual determinants, as perceived by students, on self-reported mental health, and how these impacts varied by depression, anxiety and stress.

**Methods:**

A cross-sectional survey was conducted with students attending a large university in Toronto, Canada. Participants completed a self-administered online questionnaire as part of a larger project. The questions asked about contextual determinants related to personal, interpersonal, family, social, socio-economic and political factors along with levels of depression, anxiety and stress as measured by Patient Health Questionnaire-9, Beck Anxiety Inventory and Perceived Stress Scale.

**Results:**

A total of 148 students completed the questionnaire (37 males and 111 females) with an age range of 19–54 years (median 22, IQR 21–24.8). English was reported as first language by 62.8% while 34.5% self-identified as white and 58.1% reported being born in Canada. Overall, 39.5% reported symptoms of moderate to severe depression, 23.8% reported moderate–severe anxiety and 80.3% reported moderate–severe levels of perceived stress, with no significant differences between males and females. In the final multivariate analysis, variables significantly associated with depression were grade-point-average (aOR 2.46, 95% CI 1.017–5.97), family factors (aOR 3.46, 95% CI 1.50–7.94), social factors (aOR 3.24, 1.30–8.1), self-rated health (aOR 0.34, 95% CI 0.14–0.82) and political factors (aOR 0.40, 95% CI 0.16–0.97). Anxiety was significantly associated with family factors (aOR 2.79, 1.09–7.18), socioeconomic factors (aOR 2.59, 95% CI 1.05–6.42) and age (aOR 0.33, 95% CI 0.11–0.98). The significant factors for stress were grade-point-average (aOR 2.41, 1.01–5.75) and social factors (aOR 3.87, 95% CI 1.59–9.43).

**Conclusion:**

The study found strong to moderate impact of several determinants on depression, anxiety and stress. The results suggest a need to address a variety of factors affecting students’ mental health.

*Trial registration* Parent trial: http://www.isrctn.com/ISRCTN92827275

## Introduction

Eighteen to twenty years old individuals, or youth, are vulnerable to mental illnesses. Evidence documents that 75% of mental illnesses have first-time onset during the youth years [1]. Youth is a life period where individuals experience multiple stressors due to their developmental stage, adjustment to new college environment, academic expectations, and their specific higher education program of study [2]. Increasing levels of common mental disorders like depression, anxiety and stress are becoming a global concern for young adults and college students [3]. Yet, studies with youth report considerable variation in the rates, which could be a function of differing contextual determinants.

A systematic review of depression among undergraduate students in 2013 reported a wide variation of prevalence that ranged between 10 and 84% [4]. Similar variations are notable in the Canadian studies which are recent and only a handful. In 2016, a survey of post-secondary students found that 14.8% and 18.4% were diagnosed or treated for depression and anxiety, respectively, during the past 12 months [5]. The same survey reported that 46.2% of students experienced over average stress and 14.4% tremendous stress during the past 12 months [5]. Other Canadian studies have focused on students in clinical disciplines and report higher rates of distress. In 2016, an online survey with Canadian medical students and residents reported prevalence of 41.5% for clinical levels of psychological distress while 9.2% had high to very high distress [6]. Another study with Canadian nursing students in 2013 reported prevalence of mild to severe depression, anxiety and stress as 33%, 39% and 38% respectively [7]. It’s evident that more Canadian studies are needed with post-secondary students to generate cumulative knowledge on the rates of common mental disorders and to explore the contextual contributors.

Understanding the determinants and sources of psychological distress and common mental disorders is important to reduce the burden of suffering for students, families and society. Previous studies on these disorders among students elsewhere have explored some of those factors and report associations with female gender, family dysfunction, economic hardship, lack of support, study year, coping, faculty support, lack of leisure, body image and low study satisfaction [8–14]. However, there is dearth of Canadian studies and the few available have primarily focused on students from clinical disciplines and, hence, may not represent the broader population of students [6, 7, 15–17]. Further, these studies largely focused on aspects specific to clinical experiences and gave less attention to the wider context of student life on campus, in the family and outside. Such holistic examination of contextual determinants is important for a country like Canada where 22% of residents are first generation immigrants [18] who encounter settlement challenges that may add stressors for their youth. More importantly, students’ own assessment of the negative or positive impact of these contextual determinants has not been examined to the best of our knowledge. Addressing these gaps in scholarly knowledge, we developed a framework to assess how students perceived the impact of a wide range of contextual factors related to life on campus, in the family and in the community with an aim to better understand the determinants of depression, anxiety and stress.

The primary aim of the current study was to investigate students’ perceived impact of interpersonal, family, social and socioeconomic factors along with academic progress as exposure items determining the severity of depression, anxiety and stress scores. The secondary aim was to estimate the rates of depression, anxiety and stress to continue building the evidence on the mental health needs of students, especially among ethnically diverse students in the selected Canadian university.

## Methods

The reported study was conducted in January 2017, with undergraduate students at a large university in Toronto, Canada, as part of a trial with a mindfulness online intervention; details provided elsewhere [19, 20]. The data collected for the baseline assessments is analyzed for the reported study. Ethical approval for the research undertaken was obtained from York University (Certificate e2016-345).

### Data collection procedures

Students at least 18 years of age with an active undergraduate student status were eligible to participate. The study was advertised using multiple strategies including study posters, class visits on permission of course directors, courses’ websites, and email invitations via listservs of student associations, faculty of health and faculty of liberal arts. Interested students contacted the project staff, who then applied eligibility criteria, checked students’ university identification (ID) cards with numbers and obtained informed written consent. Participating students were then asked to complete the online survey questionnaire within a 2-week time period. Students were required to enter their Student ID number for survey completion. As an incentive for participation, students had the option to: (1) receive 2% marks in a course where instructors gave permission; (2) use the Undergraduate Research Participant Pool of 1st year psychology course to obtain up to 4% of marks for volunteering 8 h in research; or (3) receive $50 honorarium.

### Measurement

Students’ self-perceived impact of contextual determinants of health on their sense of wellbeing were gathered by asking them a question about 19 factors (Table 2): “To what extent does each of the following factors have a negative impact on your sense of wellbeing?” Answer was on a scale of 0–7 where zero is “No negative impact” and 7 is “Extreme negative impact” on wellbeing. These factors were identified through focus group discussions [20] and literature review. Depression was measured using 9-item Patient Health Questionnaire (PHQ9) [21] that measures symptoms of depression on a 0–3 where zero is “Not at all” and 3 is “Nearly every day” with a maximum possible score of 27 and minimum of zero. Scores from 0–4 are considered no depression, 5–9 mild depression, 10–14 moderate depression, 15–19 moderately severe and 20–27 severe depression. Anxiety was measured using 21-item Beck Anxiety Inventory (BAI) [22, 23] that measures symptoms of anxiety on a 0–3 scale where zero is “Not at all” and 3 is “Severely-It bothered me a lot” with a maximum possible score of 63 and minimum of zero. Scores from 0–9 are considered minimal anxiety, 10–18 mild, 19–29 moderate and 30–63 severe anxiety. Generalized stress was measured using the 10-item Perceived Stress Scale (PSS) [24] that measures symptoms of stress on a 0–4 scale where zero is “Never” and 4 is “Very often” with a maximum possible score of 40 and minimum of zero. Scores from 0–13 are considered low stress, 14–26 moderate and 27–40 high stress. The survey also asked questions on participants’ socio-demographic characteristics such as age, gender, country of birth and so forth.

### Analysis and sample size

The data were first analyzed using descriptive statistics to examine the sample characteristics, means scores of exposure items and the rates of depression, anxiety and stress. Characteristics of males and female were compared using Chi square test or Mann–Whitney test. Prior to conduction of logistic regression, subscales were derived for the 19 factors based on their thematic relationship followed by internal consistency assessment using Cronbach’s alpha: interpersonal (4 items), family (3 items), social (3 items), socioeconomic (6 items), and political (2 items) with internal consistency of 0.82, 0.71, 0.53, 0.76, and 0.76, respectively; alpha for the 19-item scale was 0.88. Grade-point-average was kept on its own as a personal level item. The process of subscale derivation entailed team discussions and consensus. The subscale scores were not normally distributed therefore they were dichotomized through the median as ‘no/low’ and ‘moderate/extreme’ impact. We also tested the internal consistency of PHQ9, BAI and PSS scales in our data which gave acceptable alpha values of 0.87, 0.92 and 0.88, respectively. Prior to logistic regression, we recoded the scales for depression, anxiety and stress: PHQ9 scores as no/mild depression (score 0–9) and moderate/severe depression (score 10 and over); BAI-21 scores as minimal/mild anxiety (score 0–18) and moderate/severe anxiety (score 19 and over); and PSS scores as low stress (score 0–13) and moderate/high stress (score 14 and over). The multiple logistic regression analyses for binary outcomes were executed and the final models were evaluated for the goodness of fit using Hosmer–Lemeshow test and for multicollinearity by checking Variance Inflation Factor (VIF), which for logistic regression is recommended to be below 2.5 [25]. All analyses were conducted using Stata software. For logistic regression analysis, the data set had an adequate ratio of cases-to-variables i.e. 10 cases per predictor [26].

## Results

Out of 164 students who agreed to participate, 148 completed the survey questionnaire giving a response rate of 90%. The students came from various departments of the university but majority were from psychology (38.5%) and health (31.8%). There were 37 males (25%) and 111 females (75%). Participant age ranged from 19 to 54 years (median 22, interquartile range [IQR] 21–24.8). Most participants reported English as a first language (62.8%) and 34.5% self-identified as white, while 58.1% were born in Canada. In relation to utilization of mental health support, 41.5% of students reported having access to financial coverage (insurance) for use of private mental health counselling while 66.1% reported current or past use of mental health counseling or psychotherapy. Table 1 shows the main characteristics of the sample.

**Table 1:** Demographic and health characteristics (n = 148)

Characteristics	Number (%) or median (inter-quartile range)			p value
	All	Male	Female	
Gender				
Male	37 (25.0)	–	–	
Female	111 (75.0)	–	–	
Age group				
19–24 years	111 (75.0)	26 (70.3)	85 (76.6)	0.72
25–29 years	18 (12.2)	5 (13.5)	13 (11.7)	
30 and over	19 (12.8)	6 (16.2)	13 (11.7)	
Major				
Psychology	57 (38.5)	12 (32.4)	45 (40.6)	0.11
Health	47 (31.8)	9 (24.3)	38 (34.2)	
Other	44 (29.7)	16 (43.3)	28 (25.2)	
Born in				
Canada	86 (58.1)	21 (56.8)	65 (58.7)	0.85
Other countries	62 (41.9)	16 (43.2)	46 (41.4)	
First language				
English	93 (62.8)	21 (56.8)	72 (64.9)	0.38
Other	55 (37.2)	16 (43.2)	39 (35.1)	
Relationship status				
Single, no relationship	81 (54.7)	23 (62.2)	58 (52.3)	0.73
Single in relationship	50 (33.8)	11 (29.7)	39 (35.1)	
Married/common law	10 (6.8)	2 (5.4)	8 (7.2)	
Other	7 (4.7)	1 (2.7)	6 (5.4)	
Ethnicity				
White	51 (34.5)	13 (35.1)	38 (34.2)	0.60
South Asian	28 (18.9)	6 (16.2)	22 (19.8)	
Black	16 (10.7)	2 (5.4)	14 (12.6)	
Chinese	13 (8.8)	3 (8.1)	10 (9.0	
Other	40 (27.0)	13 (35.1)	27 (24.3)	
Self-rated health				
Poor/fair	36 (24.5)	8 (22.2)	28 (25.2)	
Good	60 (40.8)	11 (30.6)	49 (44.2)	0.18
Very good/excellent	51 (34.7)	17 (47.2)	34 (30.6)	
Obligations apart from university study^a^				
Domestic chores	91 (63.5)	20 (54.1)	71 (61.5)	0.24
Voluntary work	80 (54.1)	21 (56.8)	59 (53.2)	
Caregiving to children, parents, and/or others	34 (23.0)	5 ((13.5)	29 (26.1)	
Internship and training	19 (12.8)	8 (21.6)	11 (9.9)	
Other obligations	26 (17.6)	7 (18.9)	19 (17.1)	
Currently covered for private counselling/therapy	61 (41.5)	15 (41.7)	46 (41.4)	0.98
Ever used any counselling/therapy support, %	97 (66.1)	23 (63.9)	74 (66.8)	0.76
Years in Canada if not born there, median (IQR)	11 (4, 16)	10 (3.3, 16)	11 (4.5, 16)	0.69
Weekly paid work hours, median (IQR)	4 (0, 13)	5 (0, 14.5)	4 (0, 12)	0.70
Weekly unpaid work hours, median (IQR)	2 (0, 5)	2.5 (0.3, 7)	2 (0, 5)	0.08

^a^Percentages add up to over 100% because of overlapping obligations

### Perceived impact of contextual determinants on sense of wellbeing

Students rated 19 exposure items on a scale of 0–7 for the negative impact on their overall wellbeing (Table 2). The scores of these variables were not normally distributed therefore the medians and interquartile ranges (IQR) are reported. Several determinants seemed to have a high perceived negative impact on students’ wellbeing, such as finding a desired job (median 5, IQR 2,6), meeting family expectations (median 4, IQR 2,5), family conflict (median 4, IQR 1,5), feeling judged (median 4, IQR 1,6), paying tuition fees (median 4, IQR 2,5), social exclusion (median 4, IQR 1,5), and obtaining the desired grade-point-average or GPA (median 4, IQR 3,6). On the other hand, physical abuse, bullying, discrimination, sexual orientation, and access to health services were perceived to have little impact on their wellbeing. The perceived impact of these factors were not significantly different for males and females except for family obligations (p = 0.04), family conflict (p = 0.02) and commuting (p = 0.004) which were rated significantly higher by female students (Table 2).

**Table 2:** Perceived impact of contextual factors on sense of wellbeing using Mann–Whitney test (n = 148)

Contextual factors	Self-perceived negative impact^a^ Median (inter-quartile range)			
	All	Male	Female	p value
Interpersonal factors				
Physical abuse	1 (0, 1)	0 (0, 5)	0 (0, 1)	0.054
Bullying	1 (0, 4)	1 (0, 4)	1 (0, 4)	0.66
Feeling judged	4 (1, 6)	3 (1, 5)	4 (1, 6)	0.87
Conflict with non-family members	2 (1, 4)	2 (0, 4)	3 (1, 4)	0.71
Family factors				
Meeting family members’ expectations	4 (2, 5)	3.5 (1, 5)	4 (2, 5)	0.17
Fulfilling family obligations	2 (1, 5)	1 (0, 3.8)	3 (1, 5)	0.04
Family conflict	4 (1, 5)	3 (1, 4.8)	4 (2, 6)	0.02
Social factors				
Social exclusion	4 (1, 5)	4 (3, 6)	4 (1, 5)	0.07
Discrimination (race, ethnicity, gender)	1 (0, 4)	1 (0, 4.8)	1 (0, 4	0.85
Gender identity/sexual orientation	0 (0, 1)	0 (0, 3)	0 (0, 1)	0.44
Socioeconomic factors				
Paying tuition fees	4 (2, 5)	4 (2, 5)	4 (2, 5)	0.94
Limited access to housing and food	0 (0, 3)	0 (0, 4)	0 (0, 2)	0.78
Limited access to health services	1 (0, 4)	0 (0, 2)	1 (0, 4)	0.12
Commute to school or work	2 (1, 5)	1 (0, 2.8)	3 (1, 5)	0.004
Working conditions	0 (0,3)	0 (0, 4)	0 (0, 2)	0.28
Finding desired employment	5 (2, 6)	4 (3, 6)	5 (2, 6)	0.64
Political factors				
Local news and events	1 (0, 3)	1 (0, 3.8)	1 (0, 3)	0.78
National and global news and events	3 (1, 4)	3 (1, 4)	2 (1, 4)	0.57
Grade-point-average, GPA	4 (3, 6)	4 (3, 6)	4 (3, 5)	0.84

^a^Scale 0–7: 0 = no negative impact and 7 = extreme negative impact

### Depression, anxiety and stress

Prevalence of various levels of depression, anxiety and stress is shown in Table 3. Overall, 39.5% of students had symptoms of moderate to severe depression, 23.8% had symptoms of moderate to severe anxiety and 80.3% had symptoms of moderate to high stress. On examining the associations with the socio-demographic characteristics at bivariate level, we found that age and self-rated health were negatively correlated with scores of each of the three conditions. Voluntary work hours were negatively correlated with depression scores. There were no statistically significant differences between males and females in relation to prevalence of any of these three conditions. Similarly, being born in Canada or elsewhere, English being first language or not, length of residence in Canada (in case of those born elsewhere), self-reported ethnicity, and major field of study did not show statistically significant associations with any one of the conditions.

**Table 3:** Prevalence of depression, anxiety and stress (n = 147)

Condition	Number (%)			χ^2^, p value
	All	Male	Female	
Depression				
None	39 (26.5)	13 (36.1)	26 (23.4)	χ^2^ (3) = 2.88, p = 0.41
Mild	50 (34.0)	9 (25.0)	41 (36.9)	
Moderate	31 (21.1)	7 (19.4)	24 (21.6)	
Severe	27 (18.4)	7 (19.4)	20 (18.0)	
Anxiety				
Minimal	71 (48.3)	21 (58.4)	50 (45.1)	χ^2^ (3) = 6.7, p = 0.08
Mild	41 (27.9)	7 (19.4)	34 (30.6)	
Moderate	19 (12.9)	7 (19.4)	12 (10.8)	
Severe	16 (10.9)	1 (2.8)	15 (13.5)	
Stress				
Low	29 (19.7)	8 (22.2)	21 (18.9)	χ^2^ (2) = 0.27, p = 0.87
Moderate	86 (58.5)	21 (58.3)	65 (58.6)	
High	32 (21.8)	7 (19.4)	25 (22.5)	

Depression (PHQ9): 0–4 = none; 5–9 = mild; 10–14 = moderate; 15–27 = severe/moderately severe

Anxiety (BAI): 0–9 = minimal; 10–18 = mild; 19–29 = moderate; 30–63 = severe

Stress (PSS): 0–13 = low; 14–26 = moderate; 27–40 = high

### Multivariate analysis

Logistic regression analyses with step-wise selection were conducted with depression, anxiety or stress as binary outcome variables and nine explanatory variables: interpersonal factors; family factors, social factors, socioeconomic factors, political factors, GPA, self-rated health, age and gender. Adjusted odds ratios of variables that remained significant in the multivariate model are shown in Table 4. Compared to students with no/mild depression those with moderate to severe depression had significantly higher odds of concerns about GPA (OR 2.46), family factors (OR 3.46), social factors (OR 3.24), and lower odds of good health (OR 0.34) and concern about political factors (OR 0.40). The model explained 20% of variability in depression. Similarly, compared to students with minimal/mild anxiety, those with moderate to severe anxiety had significantly higher odds of concerns about family factors (OR 2.79), socioeconomic factors (2.59) and lower odds of being over 24 years (OR 0.33). This model explained 11% of variability in anxiety. In relation to stress, only GPA and social factors were significant; compared to low stress, moderate to high stress had significantly higher odds for GPA (OR 2.41) and social factors (OR 3.87). To double-check the effect of gender on the three conditions, we added gender to each of the final models at the end but it did not improve any of the models.

**Table 4:** Logistic regression models: contextual factors and mental health (n = 147)

	Adjusted odds ratio (95% CI)	Z, p value	Model statistics
Moderate/severe depression^a^			n = 147 Log likelihood: − 79.15 p < 0.001 R^2^ = 20% Hosmer–Lemeshow p = 0.40 Mean VIF = 1.16
GPA	2.46 (1.017–5.97)	2.0, 0.046	
Self-rated health	0.34 (0.14–0.82)	− 2.41, 0.016	
Family factors	3.46 (1.50–7.94)	2.92, 0.003	
Social factors	3.24 (1.30–8.1)	2.52, 0.012	
Political factors	0.40 (0.16–0.97)	− 2.03, 0.042	
Moderate/severe anxiety^b^			n = 147 Log likelihood: − 71.71 p < 0.001 R^2^ = 11% Hosmer–Lemeshow p = 0.90 Mean VIF = 1.15
Age 25 years and over	0.33 (0.11–0.98)	− 1.99, 0.047	
Family factors	2.79 (1.09–7.18)	2.13, 0.033	
Socioeconomic factors	2.59 (1.05–6.42)	2.06, 0.04	
Moderate/high stress^c^			n = 147 Log likelihood: − 65.17 p < 0.001 R^2^ = 11% Hosmer–Lemeshow p = 0.76 Mean VIF = 1.03
GPA	2.41 (1.01–5.75)	1.98, 0.047	
Social factors	3.87 (1.59–9.43)	2.98, 0.003	

^a^Versus ‘no/mild depression’ with PHQ9 scores 0–9

^b^Versus ‘minimal/mild anxiety’ with BAI-21 score 0–18

^c^Versus ‘low stress’ with PSS score 0–13

## Discussion

The current study advances scholarly understanding about perceived impact of a range of contextual determinants on common mental health conditions among Canadian university students. Across 19 exposure items, students rated several (i.e. family expectations, family conflict, social exclusion, feeling judged, finding desired employment, paying tuition fees and GPA) as having a moderate negative impact on their overall sense of wellbeing. In our final analysis with depression, anxiety and stress as outcomes, we grouped these determinants to family, interpersonal, social, socioeconomic and political factors with the aim of assisting policy makers in developing programs at the points of leverage. We found several of these factors were significantly associated with one or more of the three mental health conditions and are discussed in detail with suggestions for practical implications.

Family and social factors and GPA were positively associated with depression while political factors and self-rated health were negatively associated with moderate/severe depression levels. Notably, family factor was also significantly associated with moderate/sever anxiety scores. Some of these associations have been previously reported for Canadian students with depressive symptoms, such as academic workload, and limited social support [7, 27], but the impact of family-related factors has not been studied well for Canadian students. Our study makes a unique contribution by highlighting the significance of considering factors of family conflict, obligations and expectations to better understand and promote student mental health. This is an important area given that studies from other high-income countries also report such patterns. For example, the recent WHO college student survey in high income countries [9] reported significantly higher odds of common mental disorders among students with family problems, and a Finnish study [28] reported significant association of these disorders with parental divorce, death, and low parental education. Another unique aspect of our study is the inclusion of social factor which examined in a composite manner the potential negative impact ensuing from one’s sexual orientation or feelings of social exclusion and discrimination based on race, gender and ethnicity. The results show that the social factor was significantly associated with students’ moderate/severe depression and stress; a finding not previously documented through a composite measure. In terms of political factors, its negative association found in our study with depression may indicate that people with moderate/severe depression are less concerned about political news and events; perhaps a consequence of reduced interest and engagement among people with depression. The significant association of the students’ self-rated health and the moderate/severe depression scores also highlights the impact of compromised mental health on participants general health. Overall, these findings suggest a need to enhance supportive environment and activities of engagement for students to alleviate symptoms of depression and anxiety. These may include evidence-based programs on coping and stress management for depression, anxiety and stress for university students [7, 29].

Socioeconomic factor in our study (employment, working conditions, paying tuition, commuting, and access to housing and health services) was also a strong predictor of moderate/sever scores of anxiety. Conceptually, this finding is in alignment with other studies that report lack of sufficient income or financial hardship as associated with anxiety [6, 8, 13, 30, 31]. However, the composite variable in our study also included commuting, housing and access to services which could be important sources of worries for students in addition to financial hardship. The urban university from where the sample was recruited is at the north of Toronto and often called a ‘commuter’ university. These findings are suggestive that commuting adds hardship for students and investments in affordable campus residence may alleviate some of their mental stress. The university programs could also enhance efforts in experiential learning so that student’s uncertainty and stress about their future prospects and employment could be reduced; an area that needs further development and research.

Participant age was also a significant predictor of moderate/severe anxiety score in the final model. The age range was wide in our study and we found significant association of younger age (25 and below) with anxiety which might be an indication of the uncertainty younger students feel and experience about their studies and their future compared to older students who might be enrolled for continuing-education after establishing themselves in a career. However, anxiety could also be related to year-of-study (for which we did not have data) indicating more anxiety in the initial years as reported by others [8, 13, 32, 33]. In either case, the finding lends support to directing more resources towards working with younger students.

Finally, the prevalence rates of depression, anxiety and perceived stress are also notable in our study being 39.5%, 23.8%, 80.3% respectively. The prevalence of depression and anxiety are somewhat similar to those reported by Chernomas et al. [7] for Canadian students. However, the prevalence of perceived stress is high in our sample compared to the above-mentioned study (38%) but more consistent with the findings of the Canadian college survey (60.6%) [5], and similar to another Canadian study reporting rate of 82% [29]. It is possible that students experiencing stress at the time of the study were more likely to participate leading to some overestimation; nonetheless, the high rate is notable. Considering that chronic stress could affect cognitive functioning and lead to the development of various psychopathologies including burnout, depression and PTSD [34], this alarmingly high level of perceived stress in the studied sample calls for immediate attention by the educators and health planners to curb the risk of full-blown clinical disorders. In this context, our investigation on multiple contextual determinants as correlates of depression, anxiety and stress could be utilized to develop a holistic and proactive approach towards reducing personal, family and community-based stressors. Such a comprehensive program for students’ mental health would benefit from intersectoral collaborations to leverage resources especially when universities are struggling to provide mental health services [35].

The study is not without limitations. The survey was conducted as a baseline survey among students selected for a larger trial. The parent trial tested an online mindfulness intervention and the study flyer and announcements described the study as a trial on mindfulness approaches for well-being including mental and physical health; this might have increased the likelihood of participation of students experiencing stress, anxiety or depression. Such a situation might bias the results towards overestimation of prevalence rates of these conditions. However, it was not possible to avoid this due to the nature of the study. The study did not find gender differences in the prevalence of the three mental health outcomes, probably because the sample was largely composed of female students (75%) with only 37 males. Information on year of study—which could be another predictor of the mental health conditions as reported in the literature—was not collected. As any cross-sectional survey relying on self-reporting, the findings should be interpreted with information bias in mind. The sample size was also not sufficient to analyze all of the measured contextual determinants individually, and the grouping in the multivariate analysis might have diluted the effect of some of those factors. Nonetheless, the study is a first step in examining the perceived impact of multiple determinants of these mental health conditions among the university students and has produced valuable results that could help respond to existing problems.

## Conclusion

The study found high prevalence of depression, anxiety and stress among students and identified a number of significant factors associated with these conditions. Looking for the causes of mental health disorders and their solution through an ecologic perspective covering personal, interpersonal, family, social and other factors surrounding the student both inside and outside the university might give better opportunities for understanding the problem. Access to and use of the mental health services is limited, and these could not provide the solution alone. Students might need to be supported to engage in individual and group level activities to deal with stressors of university life. Further research including qualitative studies are required on stress, anxiety and depression among post-secondary students, especially in non-clinical disciplines, to explore their perspectives on the solutions and their effectiveness.

## Abbreviations

aOR: adjusted odds ratio
BAI: Beck Anxiety Inventory
GPA: grade point average
IQR: interquartile range
OR: odds ratio
PHQ9: Patient Health Questionnaire 9
PTSD: posttraumatic stress disorder
PSS: Perceived Stress Scale
VIF: Variance Inflation Factor
WHO: World Health Organization
